# Chromatin-remodeling factor CHR721 with non-canonical PIP-box interacts with OsPCNA in Rice

**DOI:** 10.1186/s12870-022-03532-w

**Published:** 2022-04-01

**Authors:** Yushun Zhang, Qiong Chen, Guanlin Zhu, Dechun Zhang, Weihong Liang

**Affiliations:** 1 College of Life Sciences, Henan Normal University, Xinxiang, China; 2 National Centre for Plant Gene Research, State Key Laboratory of Molecular Developmental Biology, Institute of Genetics and Developmental Biology, Beijing, 100101 China; 3 Hainan Yazhou Bay Seed Laboratory, Sanya, 572025 China; 4 Key Laboratory of Three Gorges Regional Plant Genetics & Germplasm Enhancement, Biotechnology Research Center, China Three Gorges University, Yichang, Hubei 443002 China

**Keywords:** Chromatin-remodeling factor, PIP box, OsPCNA, CHR721

## Abstract

**Background:**

Proliferating cell nuclear antigen (PCNA) is one of the key factors for the DNA replication process and DNA damage repair. Most proteins interacting with PCNA have a common binding motif: PCNA interacting protein box (PIP box). However, some proteins with non-canonical PIP-box have also been reported to be the key factors that interacted with PCNA.

**Results:**

Here we discovered the C terminal of a chromatin-remodeling factor CHR721 with non-canonical PIP-box was essential for interacting with OsPCNA in rice. Both OsPCNA and CHR721 were localized in the nuclei and function in response to DNA damages.

**Conclusions:**

Based on the results and previous work, we proposed a working model that CHR721 with non-canonical PIP-box interacted with OsPCNA and both of them probably participate in the DNA damage repair process.

**Supplementary Information:**

The online version contains supplementary material available at 10.1186/s12870-022-03532-w.

## Background

PCNA (Proliferating cell nuclear antigen), as an important eukaryotic DNA replication factor, involves in DNA replication, DNA repair, cell cycle control, and other metabolic processes by interacting with multiple protein factors such as DNA polymerases, flap endonuclease 1 (Fen1), DNA ligase 1 (Lig1) and so on [11–13]. PCNA can also recruit DNA damage response (DDR) factors after DNA damage for completion of DNA replication and promote post-replication [13]. PCNA participates in cell-cycle control by interacting with cell-cycle proteins, such as cyclin-CDK (cyclin-dependent kinases) complexes and CDK inhibitor p21 [7, 10, 11, 22]. To date, detailed studies of PCNA have been mainly focused on yeasts and animals, but the functions of PCNA in higher plants were not very clear. Recently, more studies of PCNA in Arabidopsis and rice have been reported. It has been reported that the homologous OsPCNA in rice played a role in the form of homotrimers [9]. OsPCNA could interact with several proteins, such as OsFEN1, OsGEN-L, and all OsRFC subunits [9, 15, 23].

Most proteins interacting with PCNA have a binding domain: PCNA interacting protein box (PIP box) with the character of QXX(L/M/I)XX(F/Y)(F/Y) (X represents any residue) [20], such as ZRANB3, FEN1, and Gen1. ZRANB3, also known as AH2, encodes a translocase and was reported to be recruited to the stalled or collapsed replication forks by interacting with PCNA and restarting stalled forks for maintaining genomic stability [4, 5, 17, 24]. Disfunction of ZRANB3 leads to increased formation of sister chromatid exchanges (SCEs) and more sensitivity to replication stress [4]. As a member of the SNF2 family, ZRANB3 contains a helicase motif, PIP box, the NZF-type zinc finger and a putative HNH-type endonuclease motif [21]. In humans, ZRANB3 is most closely related to the SIOD disorder protein SMARCAL1, and they both belong to the same subfamily with 44% identity [4, 5]. SMARCAL1 can be recruited to the DNA damage site by the single-stranded binding protein, RPA, and help to reanneal stalled replication forks [1, 3]. In rice, the closely related gene of *ZRANB3* by blasting the amino acids were *CHR721* and *CHR726,* which all belong to the SMARCAL1 subfamily, but no typical NZF-type zinc finger was detected. Although no homologous gene of *ZRANB3* were detected, the homologous gene of *SMARCAL1* in rice is *CHR721*, which was identified by map-based cloning and had functions in the reproductive development of rice probably by regulating genomic stability and cell cycle of meiosis [25].

Based on the previous work above, we are curious to investigate whether CHR721 interacts with OsPCNA although no canonical PIP box was detected in CHR721. Meanwhile, truncated proteins and point-mutations of CHR721 were used to identify the essential interacting regions between them. The discovery of linkages between OsPCNA and CHR721 will deepen the understanding of their functions.

## Results

### CHR721 interacts with OsPCNA physically in the nuclei

We previously identified a novel gene *CHR721* by map-based cloning in rice which functions probably in regulating genomic stability and cell cycle during reproductive development [25]. By blasting the amino acids in NCBI, CHR721 shows the highest identity of ZRANB3 which interacts with PCNA [4]. However, no related reports have been reported whether CHR721 interacts with OsPCNA up to the present. This hypothesis needs further investigation for deeply understanding the functional mechanism of the CHR721. Subcellular localization of OsPCNA and CHR721 showed that they were co-localized in the nuclei (Additional file 1: Fig. S1A-D). Similar to *CHR721*, higher expressions of *OsPCNA* were detected in panicles and young leaves. (Additional file 1: Fig. S1E, [25]). Furthermore, Both *OsPCNA* and *CHR721* have important roles in maintaining genomic stability and cell cycle regulation [9, 25]. These results all predicted some connections of OsPCNA and CHR721.

**Fig. 1:**
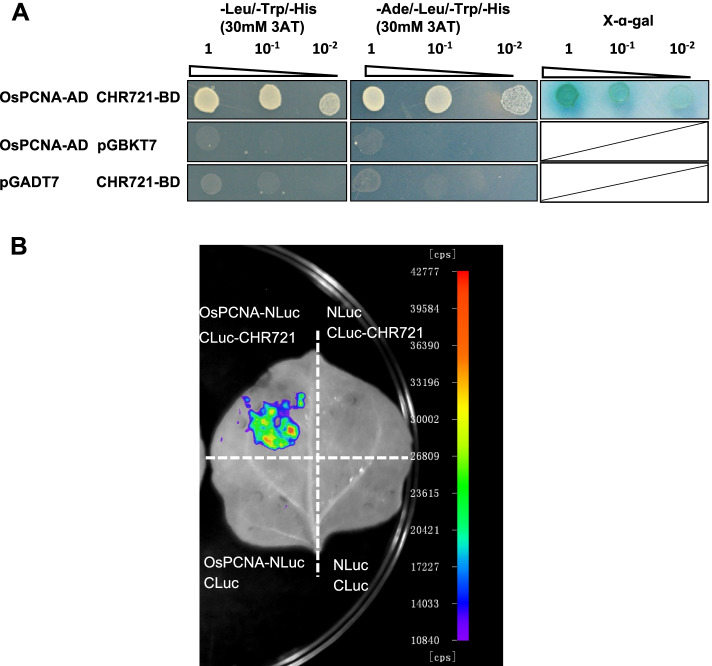
Interaction analyses of OsPCNA and CHR721 **(a)** Yeast two-hybrid assay of OsPCNA and CHR721. The co-transformed strains were spotted on SD-Leu/-Trp/-His with 30 mM 3AT and SD-Ade/-Leu/-Trp/-His with 30 mM 3AT respectively. X-α-Gal was used for detecting GAL 4-based yeast two-hybrid interactions directly. 1,10^–1^ and 10^–2^ represented the decreasing quantities of co-transformed strains spotted on SD media. **(b)** LCI assay of OsPCNA and CHR721 in *N*. *benthamiana* leaves co-infiltrated with the agrobacterial strains containing OsPCNA-NLuc and CLuc-CHR721. The negative controls were marked in the image.

To further analyze the connections of CHR721 and OsPCNA, we performed Y2H (Yeast two-hybrid) and LCI (Luciferase complementation imaging) to test whether CHR721 interacts with OsPCNA. From these Y2H experiments in strict selection conditions, it was obvious that only yeast cells harboring OsPCNA-AD and CHR721-BD could survive on SD medium lacking Ade, Leu, Trp, and His with 30 mM 3AT. This interaction was also confirmed by the X-α-Gal assay (Fig. 1A). LCI was also performed to testify the interaction between OsPCNA and CHR721 via Agrobacterium-mediated transient expression. Figure 1B showed that the expression of OsPCNA-NLuc and the empty 35S::CLuc or CLuc-CHR721 and 35S::NLuc did not show LUC complementation, whereas OsPCNA-NLuc and CLuc-CHR721 resulted in LUC complementation signal. All these experiments demonstrated that CHR721 interacted with OsPCNA proteins physically in the nuclei.

### Non-canonical PIP box in CHR721

**Fig. 2:**
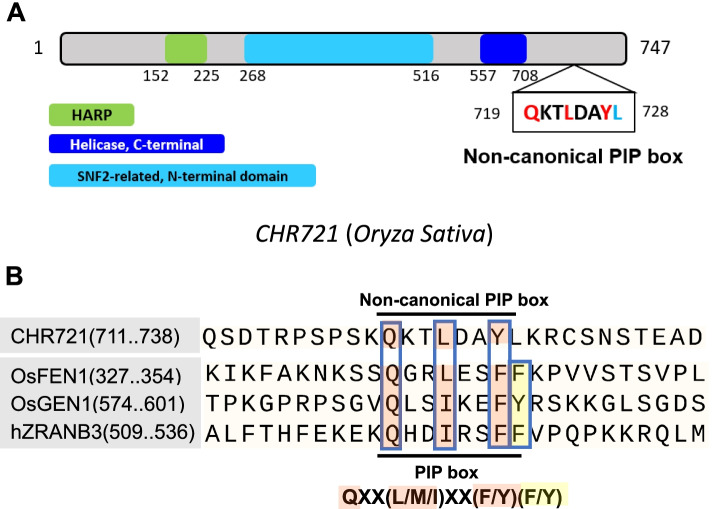
Schematic of PIP box of CHR721, hZRANB3, OsFEN1 and OsGEN1 **(a)** The Schematic of the main domains and non-canonical PIP-box of CHR721. **(b)** The characters in OsFEN1, OsGEN1, and hZRANB3 above the black line represented the PIP box. The characters of CHR721 below the black line represented non-canonical PIP-box. The characters marked in pink and yellow represented the conserved characters of the PIP box.

ZRANB3 interacts with PCNA by PIP box [4]. So, we further analyze the structure of the amino acids of CHR721. *CHR721* consists of 24 exons and the predicted CHR721 protein is 747 amino acids long and contains a putative SNF2_N domain and a putative HELICc domain (https://www.gramm ene.org/ http://rice.uga.edu/) (Fig. 2A). We selected and compared several proteins containing PIP box which interacted with PCNA, such as OsFEN1, OsGEN1, and hZRANB3. However, no canonical PIP box in CHR721 was detected by searching all the sequences of the amino acids. Only one region with ‘QKTLDAYL’ is highly identical but not the same as the PIP box. The last amino acid ‘L’ is not the consensus residue ‘F’ or ‘Y’ like OsFEN1, OsGEN1, and hZRANB3 (Fig. 2B). We also verified that OsGEN1 owning PIP box domain interacted with OsPCNA by Y2H in rice (Additional file 1: Fig. S2), which showed the same results as previous work [19].

These results indicated that CHR721 interacted with OsPCNA, although there was no canonical PIP box in CHR721.

### Regions required for the CHR721 subunits to interact with OsPCNA

**Fig. 3:**
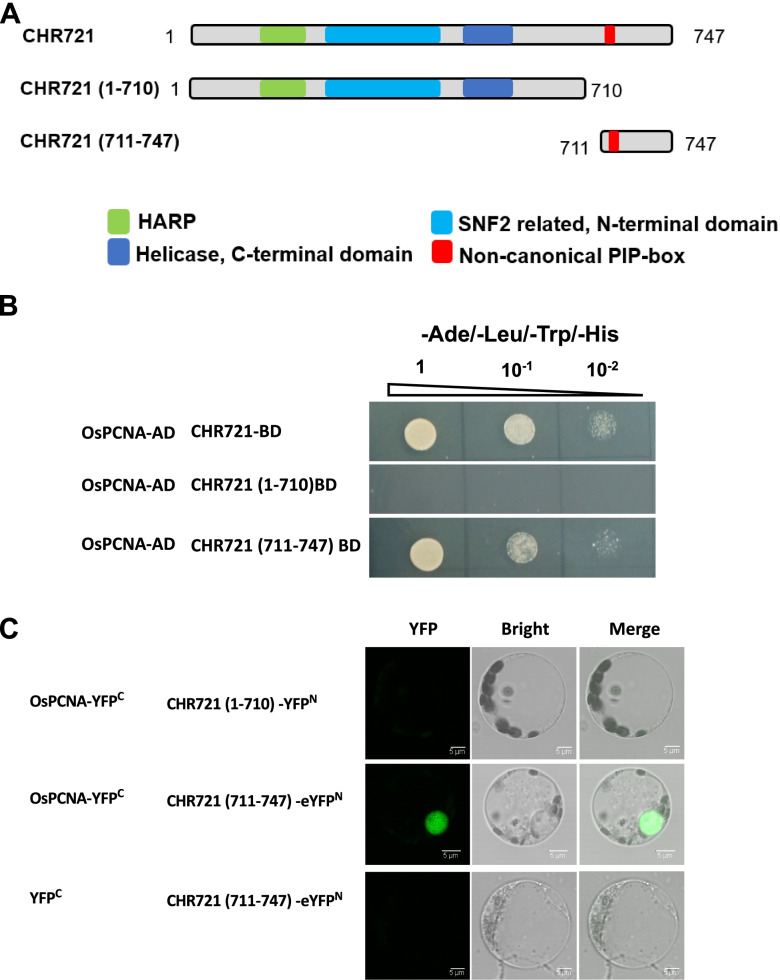
Interactions between truncated CHR721 and OsPCNA **(a)** Schematic diagrams of the regions represent the truncated CHR721. **(b)** Yeast two-hybrid assay of truncated OsPCNA and CHR721. The co-transformed strains were spotted on SD-Ade/-Leu/-Trp/-His. 1,10^–1^ and 10^–2^ represented the decreasing quantities of co-transformed strains spotted on SD media. **(c)** BIFC assay of truncated OsPCNA and CHR721. Bars: 5 μm.

To further confirm essential regions of CHR721 for interacting with OsPCNA, truncated CHR721 proteins were fused with BD (Fig. 3A). As shown in Fig. 3, the N-terminal 710 aa of CHR721 is not required for interacting with OsPCNA, CHR721 with non-canonical PIP box from 711 to 747 amino acids could interact with OsPCNA (Fig. 3A-B). Deletion of CHR721 from 1 to 710 aa did not affect the interactions between CHR721 and OsPCNA. (Fig. 3A-B). BIFC was also performed to testify this result in rice protoplast. No YFP signal was detected in cells with eYFP^C^ and CHR721(711–747)-YFP^N^, OsPCNA-YFP^C^, and CHR721(1–710)-YFP^N^. Only protoplast with OsPCNA-YFP^C^ and CHR721(711–747)-YFP^N^ could detect green signal (Fig. 3C). These results indicated that the region between 711 to 747 aa of CHR721 containing non-canonical PIP box mediated its interaction with OsPCNA.

**Fig. 4:**
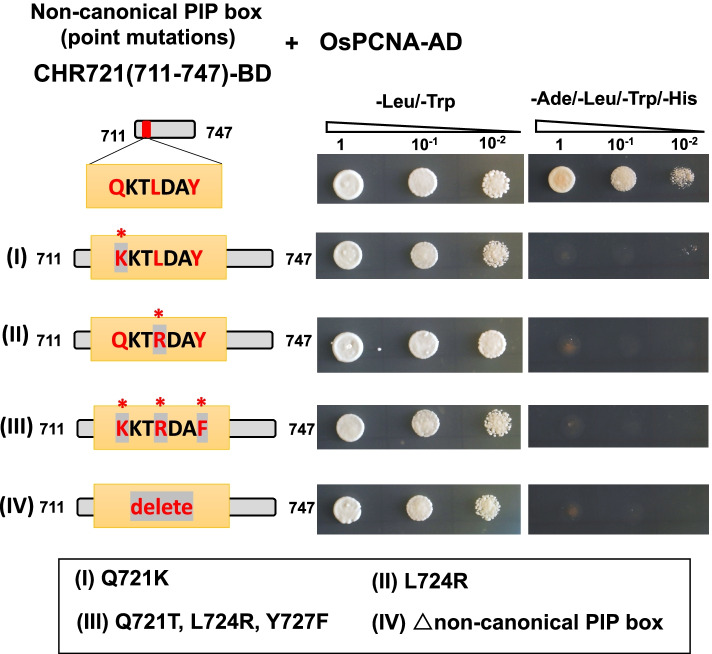
Y2H assay showing protein–protein interactions between CHR721(711–747) having different mutations in the PIP box and OsPCNA. The diagrams showed the various point-mutation sites and the deletion of the PIP box. (I) it represents Q721K. (II) it represents L724R. (III) it represents three point mutations (Q721K, L724R, and Y727F). (IV) it represents the deletion of the non-canonical PIP box

To further examine whether this interaction is mediated by the non-canonical PIP box, we designed point mutations and deletions at the conserve amino acids sites of the PIP box. Y2H results showed that the PIP-box mutations (CHR721(711–747)-PIP^Q721T^, CHR721(711–747)-PIP^L724R^, CHR721(711–747)-PIP^Q721T, L724R, Y727F^) and deletions all abrogated the interaction of CHR721(711–747) with OsPCNA (Fig. 4). In general, the conserved amino acids in the PIP box have largely affected the interactions of CHR721 and OsPCNA.

### A working Model for the functional role of OsPCNA and CHR721

**Fig. 5:**
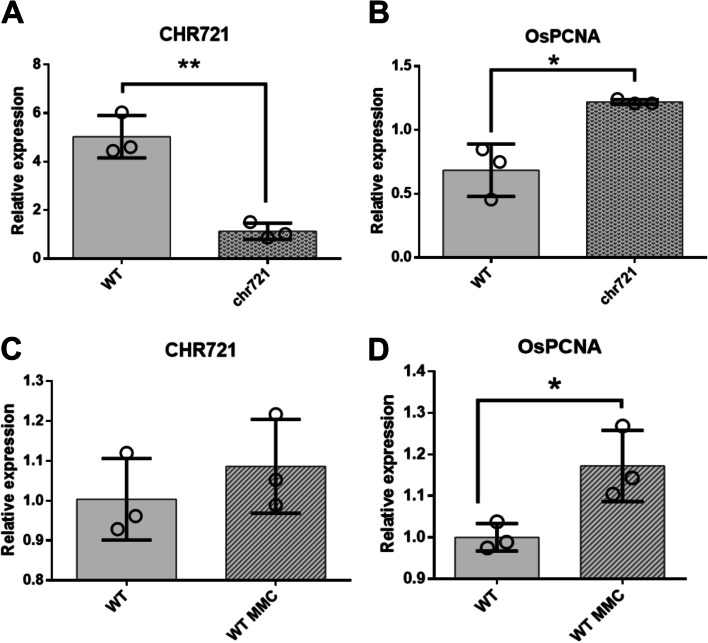
*CHR721* and *OsPCNA* expression analysis based on qRT-PCR **(**a) qRT-PCR analysis of the expression of *CHR721* at the meiotic stage between WT and *chr721* mutant. (**b**) qRT-PCR analysis of the expression of *OsPCNA* at the meiotic stage between WT and *chr721* mutant. (**c**) *CHR721* expression analysis in response to MMC treatment. (**d**) *OsPCNA* expression analysis in response to MMC treatment. Significant differences were determined by the two-tailed t-test: *P < 0.05; **P < 0.01.

Previous work detected DNA damages in *chr721* mutant at the late meiotic stage [25]. We are not confirmed whether OsPCNA participates in the DNA damage response (DDR) via direct interaction with CHR721. To testify this hypothesis, qRT-PCR revealed that the expression of *OsPCNA* in the *chr721* mutant was upregulated significantly (*P* < 0.05) at the meiotic stage of the anther (Fig. 5A-B). This result indicated that *OsPCNA* responses to DDR independently of *CHR721* at transcriptional level. We further treated the seedlings with Mitomycin C (MMC, a DNA damage agent, physically blocks DNA replication, recombination), the expression of *OsPCNA* was also significantly up-regulated after the treatment. This coincided with the above result that *OsPCNA* participates in DDR. However, the expression of *CHR721* increased a little bit (no significant, *P *> 0.05) (Fig. 5C-D). This implied that *CHR721* participating in DDR was not mainly by increasing the transcriptional level. Whether the recruitment of CHR721 (or OsPCNA) to the DNA damage site is via their interaction need furth research.

**Fig. 6:**
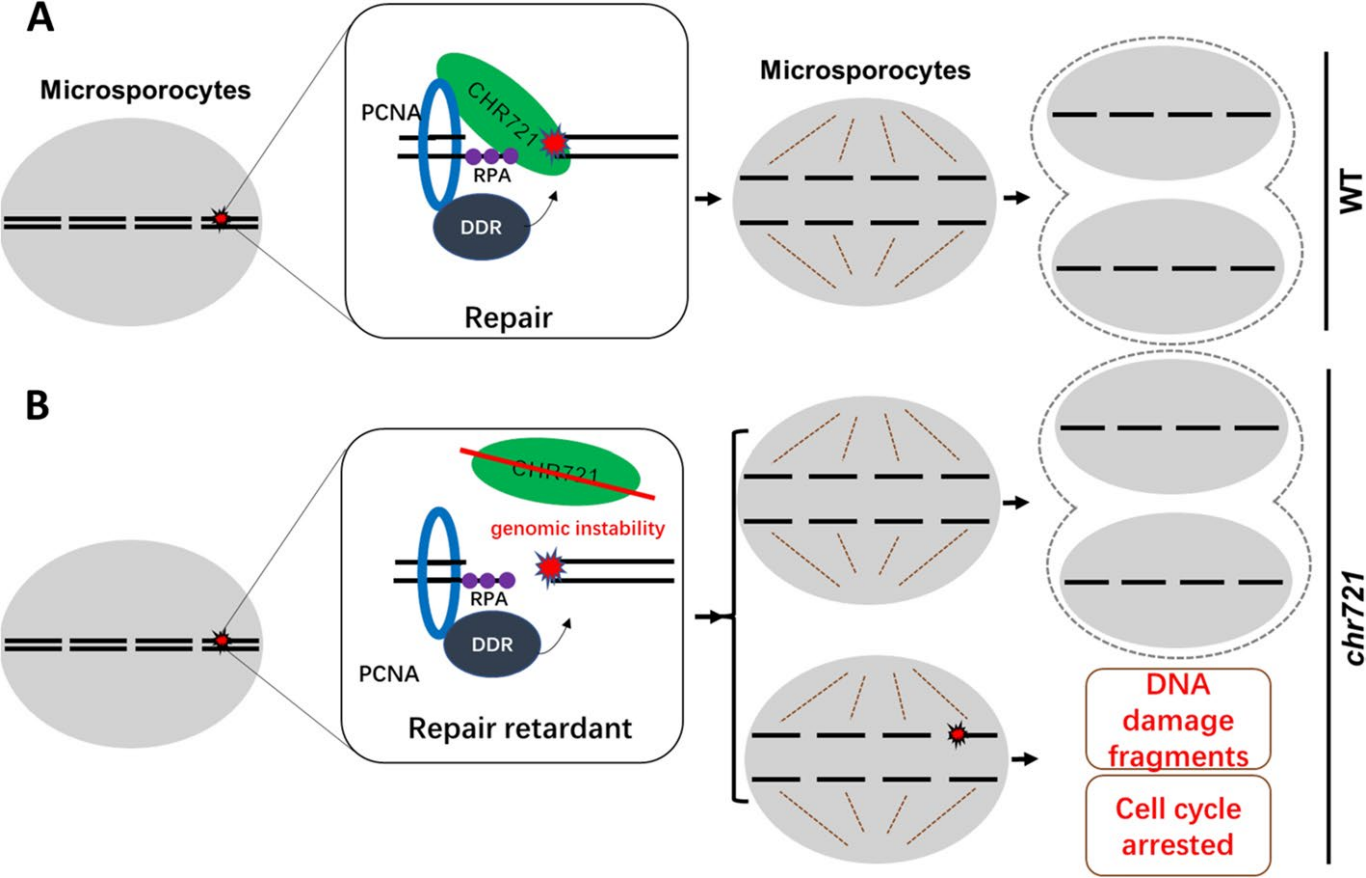
Putative working model for the functional role of OsPCNA and CHR721 **(a)** At normal conditions, the genomic DNA of microsporocytes suffered from various damages during meiosis. RPA1a recognizes the single strand and recruited CHR721 to the damage site by interacting with each other. OsPCNA with functions of recruiting DNA damage repair factors were recruited to damage sites possibly by interacting with CHR721 or other ways. After repairing, microsporocytes can divide normally. **(b)** When CHR721 lose its function, genomic instability led to repair retardants. So, we can detect DNA damage fragments and the cell cycle of microsporocytes being arrested. Some parts of cells with integrity chromatin can be divided normally.

Based on the results above and previous work, we propose a putative working model for the functional role of OsPCNA and CHR721 in the reproductive development of rice. During meiosis, various DNA damages, such as double-strand breaks (DSBs) and single-strand breaks (SSBs) occurred. In response to these DNA damages, organisms have evolved various defenses for repair. OsRPA1a binds to single-stranded DNA generated from damaged sites. In the following step, CHR721 was recruited to the DNA damage sites probably via direct interaction with OsRPA1a. CHR721 not only stabilizes the genomic DNA but also interacts with OsPCNA which can repair the DNA damage by recruiting DDR (DNA damage response) factors (Fig. 6A). When knocking down CHR721, although increased expression of OsPCNA was detected in *chr721* (Fig. 5B), the recruitment of OsPCNA to the damage sites or the activity of OsPCNA in responding to the DNA damage repair might be impaired. Loss-function of CHR721 also results in genomic instability. Thus, repairment at the damage site was impeded although other repair pathways might participate in this process. So, unrepaired or retardant repaired DNA damages resulted in the abnormal cells and cell cycle might be arrested.

## Discussion

Many partners of PCNA contain a highly conserved PCNA-binding motif: the PIP-box [8, 20]. However, more and more factors with non-canonical PIP-box have been reported to interact with PCNA and showed the diversity of the interacting motifs [14]. Here, our results revealed a novel chromatin-remodeling factor CHR721 with non-canonical PIP-box interacted with OsPCNA in rice and the C-terminal of CHR721 containing the non-canonical PIP box is essential for binding OsPCNA.

Besides the conserved structures of PCNA in different species, the functions of PCNA were still highly conserved. In rice, both OsPCNA and OsFEN-1 participate in cell proliferation [9]. OsPCNA interacts with OsGEN-L, and OsGEN1 functions in DNA repair [19, 23]. In other species, PCNA also participated in DNA replication and DNA repair by interacting with FEN1 and GEN1 [6, 16]. These indicated the conserved functions of OsPCNA compared with humans and animals.

Previous work indicated OsPCNA and CHR721 showed similar functions in genomic stability and cell cycle regulation [15, 25]. However, whether OsPCNA functions in the reproductive development just as CHR721 is not clear so far because no mutant of OsPCNA has been reported. We further decide to obtain the *ospcna* mutant by CRISPR Cas9 and this will help us to understand the function of OsPCNA.

By analyzing the 77 experimentally confirmed diversity PCNA-binding proteins [14], there are also the factors with the similar motif of CHR721 interacting with PCNA. Besides, *CHR721* and *OsPCNA* have similar tissue-specific expressions. From all these experiments and information above, a putative working model was proposed based on these results and previous work. The details of their functional mechanisms need further investigation.

## Conclusions

In this study, we discovered a novel partner of OsPCNA. Most proteins have been reported to interact with PCNA through the PIP box. Both Y2H and LCI demonstrated the interaction between OsPCNA and CHR721, although CHR721 has a non-canonical PIP box. Point mutations and deletions at the conserved amino acids sites of the PIP box abrogated the interaction, which demonstrated the essential role of the non-canonical PIP box in CHR721 on the interaction with OsPCNA. Based on the results and previous work, we proposed a putative working model that both OsPCNA and CHR721 probably participate in the DNA damage repair process. Taken together, the discovery of linkages between OsPCNA and CHR721 will be helpful for better understanding their functions.

## Materials

### Plant materials and growth conditions and treatments

The rice plants were grown in the paddy field of Henan normal university in Xinxiang under natural growth conditions. The *japonica* subspecies Tp309 was used as the wild-type, and the mutant *chr721* being supplied by Dechun Zhang (Three Gorges University, Yichang, China) was in Tp309 background throughout this study.

The spikelets at the meiotic stage were identified under the microscope after staining with acetocarmine (Solarbio, Beijing, China). Then, the fresh spikelets at the meiotic stage were collected in liquid nitrogen for the next RNA extraction.

For the MMC treatment, the surfaces of seeds were sterilized with 75% alcohol for 1 min and washed in sterilized water three times. Then, transfer the seeds to 5% NaClO for 20 min with gently shaking. After washing the seeds in sterilized water three times and air-dried naturally, seeds were grown on 1/2 MS or 1/2 MS with MMC (20 µM) (S8146, Selleck, USA) under 16/8 h of light/ dark at 28℃/ 25℃. 12-days seedlings were collected in liquid nitrogen for the next RNA extraction.

### Yeast two-hybrid assay

Yeast two-hybrid assays were performed following the protocol of Clontech, Mountain View, CA, USA. The coding sequences of *OsPCNA* and *CHR721* were cloned and fused in-frame with pGADT7 (the insertion sites: NdeI and BamHI) and pGBKT7 (the insertion sites: NdeI and BamHI) respectively. The truncated cDNAs of *CHR721* were amplified and cloned into pGBKT7. The DNA fragments including point mutations in the PIP box of CHR721 were synthesized and fused in-frame with pGBKT7 respectively. All the constructed vectors were confirmed by sequencing. The cells of yeast strain AH109 were transformed and plated on synthetic media. The β-galactosidase assays were performed according to the protocol of the MATCHMAKER kit (Clonetech). Primers used for cDNA amplification were listed in Additional file 2: Table S1.

#### BIFC

Protoplast-based bimolecular fluorescence complementation (BIFC) was performed to test protein interactions, following the protocol with little modification [18]. Protoplasts are produced from 10 to 14-day-old rice seedlings on 1/2 MS medium at 28℃ with a 12 h-light/ 12 h-dark cycle. Stem and sheath were cut into approximately 0.5 mm strips and which were transferred into enzymatic digestion solutions (0.6 M mannitol, 10 mM MES, pH5.7, 1.5% cellulase RS, 0.75% Macerozyme, 0.1% BSA, 3.4 mM CaCl2-2H2O, 14.4 mM β-Mercaptoethanol, 100 mg/ml Ampicillin) immediately. After 4 h with gentle shaking, the protoplasts were collected by filtration through 40 μm nylon meshes. The collecting protoplasts were washed in W5 solution (154 mM NaCl, 125 mM CaCl2, 5 mM KCl, 2 mM MES pH5.7) twice and suspended in MMg solution (precooling, 0.6 M mannitol, 15 mM MgCl2, 4 mM MES pH 5.7). The vectors containing encoding genes fused with YFP^C^ (the insertion sites: XbaI and XhoI) and YFP^N^ (the insertion sites: XbaI and BamHI) were co-transferred to the protoplasts by PEG-mediated (40% PEG) transformation and incubated at 28℃ for 12 to 16 h. The YFP signal will be detected by confocal if the two proteins interact. Primers used for BIFC were listed in Additional file 2: Table S1.

### LCI (Firefly luciferase complementation imaging) assay

The LCI assay was performed according to reference [2] with slight modifications [25]. The coding sequences of *OsPCNA* and *CHR721* were cloned into pCAMBIA1300:nLUC and pCAMBIA1300:cLUC respectively (the insertion sites: KpnI and SalI). The positive Agrobacterium (GV3101 strain) containing the constructed plasmid was cultured, collected and suspended with suspension (10 mM MES, 10 mM MgCl2, 200µmAS). Equal volumes of Agrobacterium suspensions carrying the indicated constructs were infiltrated into *N. benthamiana* leaves. After infiltration, plants were placed at 23 °C for 48 to 72 h under 16-h light/8-h dark conditions. Then, the leaves were sprayed with 0.5 mM luciferin and placed in darkness for 5 min. After that, the low-light-cooled CCD imaging apparatus (NightOWL II LB983 with Indigo software) was used to capture the LUC image [2]. Primers used for LCI were summarized in Additional file 2: Table S1.

### Subcellular localization

The CDS of *OsPCNA* was amplified without its stop codon and the amplification product was fused to GFP. The mCherry was fused to the C terminal of CHR721 without its stop codon. The two vectors containing 35S::OsPCNA-GFP and 35S::CHR721-mCherry were used for the PEG-mediated transformation to the rice protoplasts. The details of the PEG-mediated transformation to the rice protoplasts were according to the method of BIFC. The fluorescence signals were imaged using a laser scanning confocal microscope (Olympus). Primers used for subcellular localization were summarized in Additional file 2: Table S1.

### qRT-PCR analysis

Total RNA was isolated using Trizol reagent (Invitrogen, USA). First-strand cDNA was synthesized from 2 μg of total RNA using a SuperScript III Reverse Transcriptase kit (Invitrogen, USA). qRT-PCR was performed using SYBR Green real-time PCR master mix (QPK-201, TOYOBO, Japan), and performed using a Light Cycle®96 Instrument (Roche). The thermal profile was as follows: 95℃, 30 s, 1 cycle; 95℃, 5 s, 58℃, 10 s, 72℃, 15 s, 40 cycles. OsActin1 (LOC_Os03g50885) was used as an internal control. The relative expression was calculated from the threshold cycle (CT) values using the 2^(-△△Ct) method. The figures represent the means ± SE of three replicates. Significant differences were determined by the two-tailed t-test in GraphPad Prism 6. The primers used are listed in Additional file 2: Table S1.

## Supplementary Information

Below is the link to the electronic supplementary material.

**Additional file 1**: **Fig. S1** Subcellular localization and expression pattern analysis of OsPCNA in rice. (a) The GFP signal shows the localization of OsPCNA in rice protoplasts. (b) The mCherry signal shows the localization of CHR721 in rice protoplasts. (c) The image of the bright light under confocal. (d) Merge image of A, B, and C. (e) Expression pattern analysis of OsPCNA in different organs.**Fig. S2 **Interaction analyses of OsGEN1 and OsPCNA. (a) Schematic of PIP box of OsGEN1; (b)Interaction analyses of OsGEN1 and OsPCNA by Y2H assay. The co-transformed strains were spotted on SD-Leu/-Trp and SD-Ade/-Leu/-Trp/-His respectively. 1,10^-1^ and 10^-2^ represented the decreasing quantities of co-transformed strains spotted on SD media. OsPCNA-AD with pGBKT7 and pGADT7 with OsGEN1-BD are the negative controls.

**Additional file 2: Table S1**: Primers used in this paper

**Additional file 3**: List of protein sequences used in the present study.

## Data Availability

The plant materials involved in this study are available from the corresponding author for research use only. All of the data supporting our research findings are included in this article (Methods section and supplementary information files).

## Abbreviations

**PCNA**: Proliferating cell nuclear antigen
**PIP box**: PCNA interacting protein box
**Fen1**: Flap endonuclease 1
**Lig1**: DNA ligase 1
**DDR**: DNA damage response
**CDK**: Cyclin-dependent kinases
**SCEs**: Sister chromatid exchanges
**Y2H**: Yeast two-hybrid
**LCI**: Luciferase complementation imaging
**MMC**: Mitomycin C
**DSBs**: Double-strand breaks
**SSBs**: Single-strand breaks
