# Supplementary figures and images for: Chromatin-remodeling factor CHR721 with non-canonical PIP-box interacts with OsPCNA in Rice
Source: BMC Plant Biol. 2022 Apr 1;22:164. doi: 10.1186/s12870-022-03532-w (PMC8974069; doi:10.1186/s12870-022-03532-w)

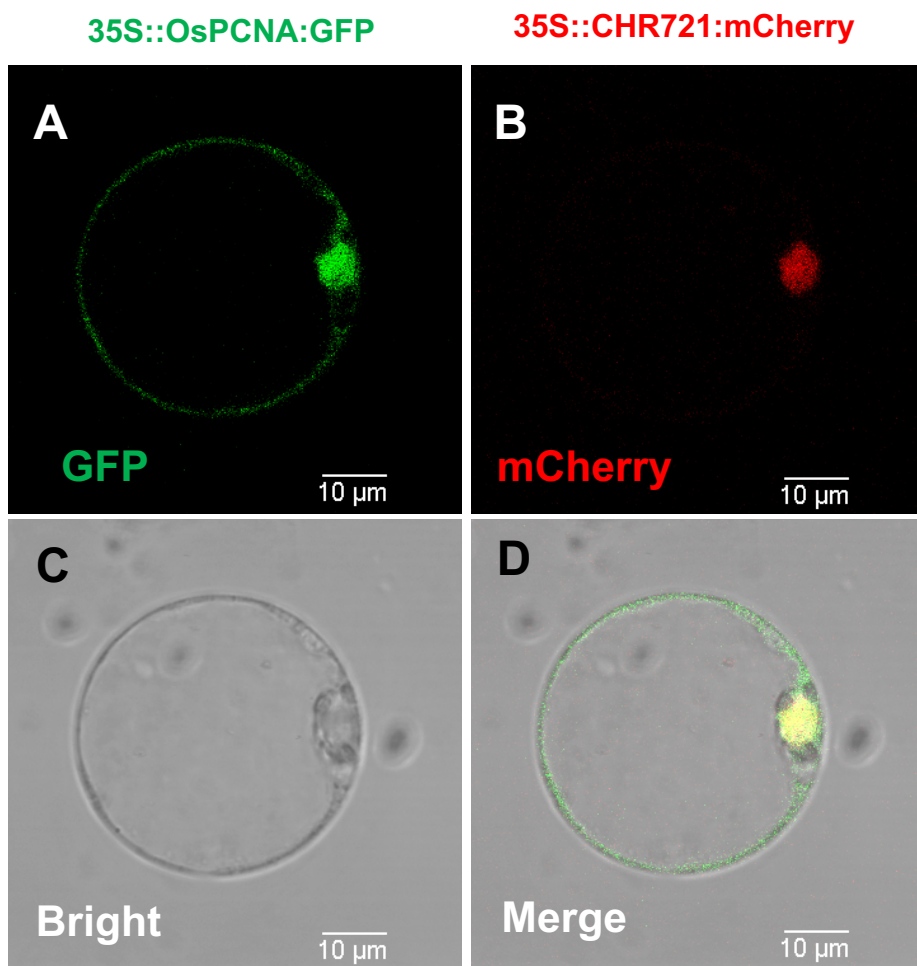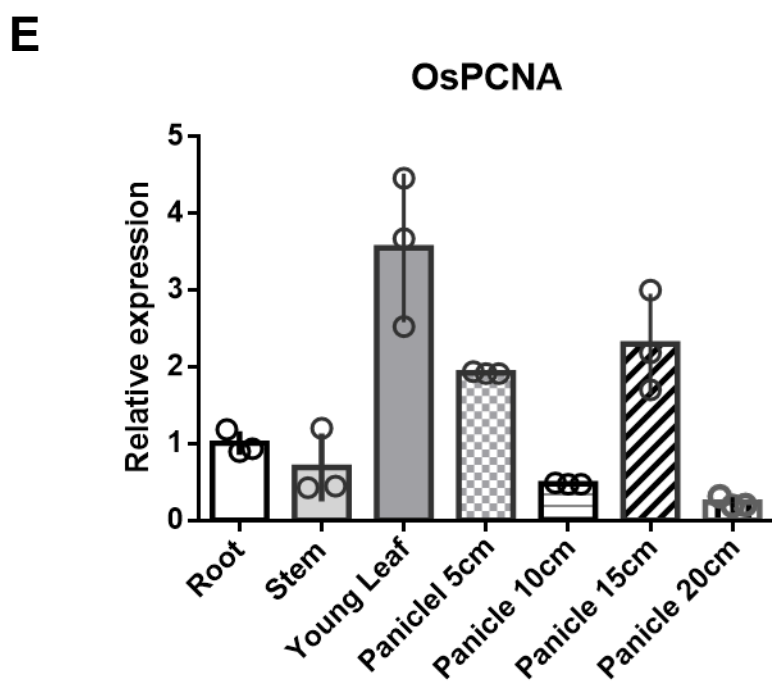

Supplemental Figure 1

**A**

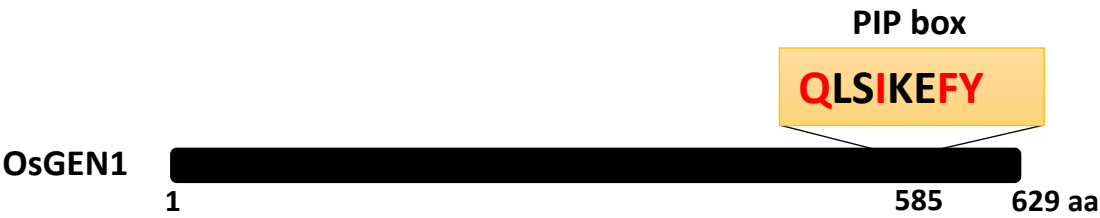

**B**

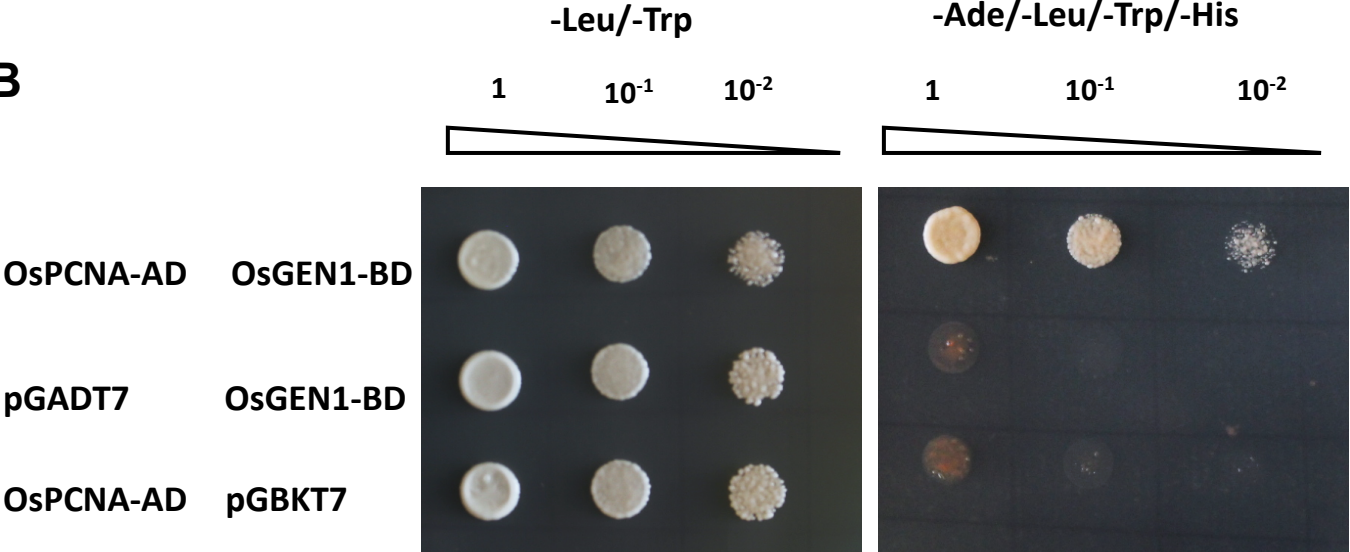

Supplemental Figure 2

Supplement: Supplementary file 1 — Additional file 1: Fig. S1 Subcellular localization and expression pattern analysis of OsPCNA in rice. (a) The GFP signal shows the localization of OsPCNA in rice protoplasts. (b) The mCherry signal shows the localization of CHR721 in rice protoplasts. (c) The image of the bright light under confocal. (d) Merge image of A, B, and C. (e) Expression pattern analysis of OsPCNA in different organs.Fig. S2 Interaction analyses of OsGEN1 and OsPCNA. (a) Schematic of PIP box of OsGEN1; (b)Interaction analyses of OsGEN1 and OsPCNA by Y2H assay. The co-transformed strains were spotted on SD-Leu/-Trp and SD-Ade/-Leu/-Trp/-His respectively. 1,10-1 and 10-2 represented the decreasing quantities of co-transformed strains spotted on SD media. OsPCNA-AD with pGBKT7 and pGADT7 with OsGEN1-BD are the negative controls. [file 12870_2022_3532_MOESM1_ESM.pdf]
