# Supplementary material for: Chromatin-remodeling factor CHR721 with non-canonical PIP-box interacts with OsPCNA in Rice
Source: BMC Plant Biol. 2022 Apr 1;22:164. doi: 10.1186/s12870-022-03532-w (PMC8974069; doi:10.1186/s12870-022-03532-w)
Supplement: Supplementary file 2 — Additional file 2: Table S1: Primers used in this paper [file 12870_2022_3532_MOESM2_ESM.docx]

Table S1, primers used in this paper

| Name | Sequence (5’-3’) |
| --- | --- |
| nLUC-PCNA-F | cgggggacgagctcggtaccATGTTGGAGCTGAGGCTT |
| nLUC-PCNA-R | gcgtacgagatctggtcgacCGACTTCATTTCCTCATCTTCTT |
| cLUC-Snf2-F | acgcgtcccggggcggtaccATGATCCGTTGGGTACAAGG |
| cLUC-Snf2-R | cgaaagctctgcaggtcgacGAACCTAGGGTTCTTGAGCT |
|  |  |
| OsPCNA/GFP-F | TACAGAACCGACGAACTAGTATGTTGGAGCTGAGGCTTG |
| OSPCNA/GFP-R | GCCCTTGCTCACCATCGACTTCATTTCCTCATCT |
| 35S+PCNA-GFP-F | AGATTGTCGTTTCCCGCCTT |
| 35S+ PCNA-GFP-R | ATTGCCAAATGTTTGAACGATC |
| mcherry-SmaI | TCCCCCGGGATGGTGAGCAAGGGCGAG |
| mcherry-SacI | cgagctcCTACTTGTACAGCTCGTCCA |
|  |  |
| NE-SNF-N-1F | GAGAACACGGGGGACTCTAGAATGATCCGTTGGGTACAAGG |
| NE-SNF-N-1R | GACAGTACTATCGATGGATCCGGACACATCCAGCGTTTTC |
| NE-SNF-C-1 F | GAGAACACGGGGGACTCTAGAATGCAGAGCGACACTAGGCCC |
| NE-SNF-C-1R | GACAGTACTATCGATGGATCCGAACCTAGGGTTCTTGAGC |
| CE-PCNA-F | GAGAACACGGGGGACTCTAGAATGTTGGAGCTGAGGCTTGT |
| CE-PCNA-R | CCCGGGAGCGGTACCCTCGAGCGACTTCATTTCCTCATCT |
|  |  |
| CHR721N-1BDF | GGAGGACCTGCATATGATGATCCGTTGGGTACAAGG |
| CHR721 N-1BDR | CGCTGCAGGTCGACGGATCCCGGACACATCCAGCGTTTTC |
| CHR721C-1BDF | GGAGGACCTGCATATGCAGAGCGACACTAGGCCC |
| CHR721C-1BDR | CGCTGCAGGTCGACGGATCCCGAACCTAGGGTTCTTGAGC |
| CHR721-BDF | GAGGACCTGCATATGATGATCCGTTGGGTACAAG |
| CHR721-BDR | CGCTGCAGGTCGACGGATCCCGAACCTAGGGTTCTTGAGC |
| OsPCNA-ADF | ACGTACCAGATTACGCTCATATGATGTTGGAGCTGAGGCTT |
| OsPCNA-ADR | TGCAGCTCGAGCTCGATGGATCCCGACTTCATTTCCTCATCTTCTT |
| OsGEN1-BDF | CAGAGGAGGACCTGCATATGATGGGGGTGGGGGGAAGC |
| OsGEN1-BDR | GCTGCAGGTCGACGGATCCCGTCGAAGAGGAGGCGTCGTC |
|  |  |
| qOsPCNA F | GGAGACACTGTTATCATCTCGG |
| qOsPCNA R | CTCTATGATTGTAGCATCTTCTGG |
| qCHR721 F | TCAAGGCAGCAGTGTTATCCA |
| qCHR721 R | TCACCGAAGAAACCTGACCA |
| qOsActin1F | ACATCGCCCTGGACTATGACCA |
| qOsActin1R | GTCGTACTCAGCCTTGGCAAT |
