# Supplementary material for: Chromatin-remodeling factor CHR721 with non-canonical PIP-box interacts with OsPCNA in Rice
Source: BMC Plant Biol. 2022 Apr 1;22:164. doi: 10.1186/s12870-022-03532-w (PMC8974069; doi:10.1186/s12870-022-03532-w)
Supplement: Supplementary file 3 — Additional file 3: List of protein sequences used in the present study. [file 12870_2022_3532_MOESM3_ESM.docx]

Additional File S3: List of sequences used in the present study.

**Protein sequences**

>CHR721│Os07g0636200│*Oryza Sativa*│(From http://rice.uga.edu/)

MIRWVQGRRGYYSPSSENPQIPSPARARALNFLGEEKISPWREPRPRMAGLGGGWGGGYDDDDDWGLSAEQLDQLERDAYRKLAERKASSSAASTATSPLPSAAYSPVKNSHHHPASRVSQESCFGKVESLSPSRLSQPNASGNAVNNSQGNMSKVSVHLFLHSTGVIAAKFQYHQKLVDAVHKIPKASWNGKERVWMFPHSSLSVAEEVLSTVPGIAVEVQKLDPLVKRALTASLYAGDLRDLYGKIPTDVESKLMPFQREGVRFALQHGARTLIADEMGLGKTLQAIAVASCLHDAWPVLVISPSSLRLHWASMIQHWLNIPTEDILVVLPQTGGSNKAGYRLVYSNTKGDFNLDGVFNVISYDVVPKIKDMLLDLDFKIVIADESHFLKNAQAKRTMHSLPVLQKAKYVVLLSGTPALSRPIELFTQLQALYPTVYKNVNEYGNRYCKGGFFGLYQGASNHEELHNLMKATVMIRRLKKDVLSQLPVKRRQQAFLDLSEKEMRHIRALFHELETVKIKIQSCDSQETMDSLKFAQKNLINKIYNDSAEAKIPAVLDYLGTIIEAECKFLIFAHHQSMLEAIHQHLLKKKVKCIRIDGQTPVPVRQTLVTDFQNKDDIKAAVLSIKAGGVGLTLTAASTVIFAELSWTPGDLIQAEDRAHRIGQVSSVNIYYLLANDTVDDIIWDVVQGKLENLGQMLDGQEKTLDVSQSDTRPSPSKQKTLDAYLKRCSNSTEADQPKLKNPRF

>OsGEN1│Os09g0521900│*Oryza Sativa*│(From http://rice.uga.edu/)

MGVGGSFWDLLKPYARHEGAGYLRGRRVAVDLSFWVVSHSAAIRARSPHARLPHLRTLFFRTLSLFSKMGAFPVFVVDGQPSPLKSQVRAARFFRGSGMDLAALPSTEAEASADALVQPRNAKFTRYVEDCVELLEYLGMPVLRAKGEGEALCAQLNNQGHVDACITSDSDAFLFGAKTVIKVLRSNCKEPFECYNMADIESGLGLKRKQMVAMALLVGSDHDLHGVPGFGPETALRFVQLFDEDNVLAKLYEIGKGVYPFIGVSAPNIDDLPSPSTKSLPRARSPHCSHCGHPGNKKNHIKDGCNFCLVDSLENCVEKPAGFICECPSCDKARDLKVQRRNENWQIKVCKRIAAETNFPNEEIINLYLNDDNLDNENGVPLLTWNKPDMEILVDFLSFKQNWEPAYIRQRMLPMLSTIYLREMASSQSKSFLLYDQYKFHSIQRIKIRYGHPYYLVKWKRVTRSMISNDPPSKQTELEGKNDKVEVLDGDDEVVDEEEEEPTMISETTELLDEPDVPQVLDDDKDCFLLTDEDIELVNAAFPDEAQRFQEEQRLKEAKSIARKSKLNVAGFETPKGPRPSGVQLSIKEFYRSKKGLSGDSGKDGSRKSSDVDLSKNLPKSVRRRLLFD

>OsFEN1│Os05g0540100│*Oryza Sativa*│(From http://rice.uga.edu/)

MGIKGLTKLLADNAPKAMKEQKFESYFGRRIAVDASMSIYQFLIVVGRTGMETLTNEAGEVTSHLQGMFNRTIRLLEAGIKPVYVFDGKPPDLKKQELAKRYSKREDATKELTEAVEEGDKDAIEKFSKRTVKVTKQHNEECKRLLRLMGVPVVEAPCEAEAECAALCINDMVYAVASEDMDSLTFGAPRFLRHLMDPSSKKIPVMEFEVAKVLEELELTMDQFIDLCILSGCDYCDSIKGIGGQTALKLIRQHGSIESILENINKDRYQIPEDWPYQEARRLFKEPNVTLDIPELKWNAPDEEGLVEFLVKENGFNQDRVTKAIEKIKFAKNKSSQGR

>hZRANB3│*Homo sapiens* (From https://www.uniprot.org/uniprot/Q5FWF4#sequences)

MPRVHNIKKSLTPHISCVTNESDNLLDFLPDRLRAKLLPFQKDGIIFALKRNGRCMVADEMGLGKTIQAIGITYFYKEEWPLLIVVPSSLRYPWTEEIEKWIPELSPEEINVIQNKTDVRRMSTSKVTVLGYGLLTADAKTLIDALNNQNFKVVIVDESHYMKSRNATRSRILLPIVQKARRAILLTGTPALGRPEELFMQIEALFPQKFGRWTDYAKRYCNAHIRYFGKRPQWDCRGASNLNELHQLLSDIMIRRLKTEVLTQLPPKVRQRIPFDLPSAAAKELNTSFEEWEKIMRTPNSGAMETVMGLITRMFKQTAIAKAGAVKDYIKMMLQNDSLKFLVFAHHLSMLQACTEAVIENKTRYIRIDGSVSSSERIHLVNQFQKDPDTRVAILSIQAAGQGLTFTAASHVVFAELYWDPGHIKQAEDRAHRIGQCSSVNIHYLIANGTLDTLMWGMLNRKAQVTGSTLNGRKEKIQAEEGDKEKWDFLQFAEAWTPNDSSEELRKEALFTHFEKEKQHDIRSFFVPQPKKRQLMTSCDESKRFREENTVVSSDPTKTAARDIIDYESDVEPETKRLKLAASEDHCSPSEETPSQSKQIRTPLVESVQEAKAQLTTPAFPVEGWQCSLCTYINNSELPYCEMCETPQGSAVMQIDSLNHIQDKNEKDDSQKDTSKKVQTISDCEKQALAQSEPGQLADSKEETPKIEKEDGLTSQPGNEQWKSSDTLPVYDTLMFCASRNTDRIHIYTKDGKQMSCNFIPLDIKLDLWEDLPASFQLKQYRSLILRFVREWSSLTAMKQRIIRKSGQLFCSPILALEEITKQQTKQNCTKRYITKEDVAVASMDKVKNVGGHVRLITKESRPRDPFTKKLLEDGACVPFLNPYTVQADLTVKPSTSKGYLQAVDNEGNPLCLRCQQPTCQTKQACKANSWDSRFCSLKCQEEFWIRSNNSYLRAKVFETEHGVCQLCNVNAQELFLRLRDAPKSQRKNLLYATWTSKLPLEQLNEMIRNPGEGHFWQVDHIKPVYGGGGQCSLDNLQTLCTVCHKERTARQAKERSQVRRQSLASKHGSDITRFLVKK
